# Split application of reduced nitrogen rate improves nitrogen uptake and use efficiency in sweetpotato

**DOI:** 10.1038/s41598-019-50532-2

**Published:** 2019-10-01

**Authors:** Xiangbei Du, Min Xi, Lingcong Kong

**Affiliations:** 1 Crop Research Institute, Anhui Academy of Agricultural Sciences, Hefei, 230031 Anhui Province P.R. China; 2 Rice Research Institute, Anhui Academy of Agricultural Sciences, Hefei, 230031 Anhui Province P.R. China

**Keywords:** Plant sciences, Solid Earth sciences

## Abstract

Splitting nitrogen (N) application is beneficial for enhancing sweetpotato growth and promoting optimum yields under reduced N rates; however, studies concerning how split N can affect sweetpotato N dynamics and utilization are limited. Field experiments were conducted from 2015 to 2016 to determine how split N application affects sweetpotato N uptake and N use efficiency (NUE) under a reduced N rate. Two cultivars (Xushu 22 and Shangshu 19) were planted under four N treatments, a conventional basal application of 100 kg N ha^−1^ (100:0), a basal application of 80 kg N ha^−1^ (80:0), two equal split applications of 80 kg N ha^−1^ (basal and 35 days after transplanting, 40:40) and a N omission treatment (N0). Data from two years revealed that sweetpotato yields decreased at a reduced 20% N rate with a basal application (80:0); however, the reduced 20% N rate with a split application (40:40) significantly increased the yield by 16.6–19.0%. Although the 80:0 treatment decreased sweetpotato N uptake, the 40:40 treatment increased the N uptake by increasing the N uptake rate and prolonging the duration of the fast N uptake phase. In comparison to the basal application, the split N application used N more efficiently, showing consistently higher levels of agronomic use efficiency, recovery efficiency, physiological efficiency and partial factor productivity. NUEs under split N improved due to increased N uptake during the middle and late growth stages and a higher N partition ratio to the storage root. The above results indicate that split N application provides better N for crop developmental stages and is recommended as an alternative approach to simultaneously increasing storage root yield and NUE under a reduced N rate in sweetpotato production in China.

## Introduction

Sweetpotato (*Ipomoea batatas* L.) is the world’s seventh most important food crop, and is a major contributor to the energy and phytochemical source of nutrition^1,2^. It is also a subsistence crop and has tremendous economic and social importance in developing countries^3^. World production is centered in Southeast Asia, with China being the largest producer of sweetpotato in the world. The cultivated area of sweetpotato in China, approximately 6.6 million hectares, accounts for 70% of the total area in the world. At present, the proportions of sweet potato processing, fresh food and forage use in China are 55%, 30% and 10%, respectively^4^. The global demand for sweetpotato is on the rise due to its high nutritional value. The contribution of sweetpotato to health from its high nutrient content and anticarcinogenic and cardiovascular disease prevention properties has been acknowledged^5^. The consumption of sweetpotatoes is gradually increasing worldwide; however, little research has been carried out on a reasonable fertilization technique for its production.

Nitrogen (N) is an essential nutrient for sweetpotato growth. To ensure high yields, application of more N than actual need is practiced by most sweetpotato farmers, especially in China. Recent studies have shown that the storage root yield does not increase as rapidly as prior to fertilizer despite the increased fertilizer application rate, and this scenario primarily due to the excessive and inappropriate timing of fertilizer application^6,7^. Over–application of N fertilizer is a serious problem in current high yielding sweetpotato production, leading to early senescence, overgrowth, unbalanced source–sink relationships, less storage root yield and low N use efficiency (NUE)^7^, in addition to substantial N losses and the creation of environmental pollution.

According to previous studies^6–13^, the application of 75–135 kg N ha^−1^ was reported to be most suitable with respect to the yield and quality of the sweetpotato. Traditionally in China, the usual N fertilizer rate for sweetpotato has been 90–200 kg ha^−1^, which is far beyond the amount crop needed and hence causes a reduction in yield and severe negative environmental impacts^7^. Therefore, optimized N management is imperative to improve NUE and reduce the negative impacts on the environment. Previous research found that N fertilizer could be reduced by approximately 12%–50% of the conventionally applied rates without sacrificing grain yield in rice^14,15^. However, reduced N application rates for sweetpotato have rarely been studied. More importantly, conventional N fertilizer for sweetpotato is applied at the time of field fumigation in China. However, the majority of N uptake by crops occurs during the later growing season when the crop is growing rapidly^11,12^. The N fertilizer applied before transplanting needs to stay in the soil for almost 30 d to contribute to the nutrition of the sweetpotato crop, increasing the risk of losing N through volatilization, immobilization, denitrification and/or leaching^16^. Obviously, a large amount of N fertilizer prior to transplanting results in poor synchronization between the soil N supply and the plant demand, resulting in a high soil inorganic N concentration before the happen of rapid plant N uptake^11,12^. This maximized the risks of N losses and led to a large decrease in NUEs^17,18^.

Numerous studies have sought to improve NUE by developing N management practices based on a better synchronization between the supply of N and plant demand. Many different measures have been suggested to increasing crop NUE, such as using the optimal rate, application time, and application method for matching N supply with plant demand. N fertilizer rates and application timing are a decisive factor in the obtaining of high yields^19–22^. Split N application at planting and later during crop growth can be used to shortening the time that inorganic N is present in the soil solution before crop uptake^23^. Recent studies have shown that in comparison to single N application, split applications of N fertilizer result in higher recovery efficiency and higher grain yields^22,24^. Split N application is the most widely adopted method for grain production worldwide^25^. Split application of N at planting and 30 days after planting (DAT) contributes to high storage root yields^26,27^. In addition, delayed N application is favorable for storage root formation^12^. Previous studies have mostly focused on crop productivity, whereas few studies have investigated split N effects on N status, N uptake and use efficiency of sweetpotato. The relationship between NUE and N uptake as affected by split N application is still unclear.

The purpose of this study was to improve N management in sweetpotato to enhance NUE and storage root yield without increasing N fertilizer application. On the basis of previous results, we hypothesized that split N application under a reduced rate would enhance plant N uptake, N harvest index (NHI), and NUE and would thereby increase storage root yield. Field experiments were established to analyze the mechanisms through which split N application affects sweetpotato N dynamics and utilization on reduced N. Objectives of this study were (a) to clarify the effect of split N application with a reduced N rate on sweetpotato N dynamics and (b) to compare the N uptake and use efficiency of different N treatments. We believe that the findings of this study will provide theoretical and practical bases for sweetpotato N management in China and abroad.

## Results

### Storage root yield response to nitrogen treatment

**Figure 1:**
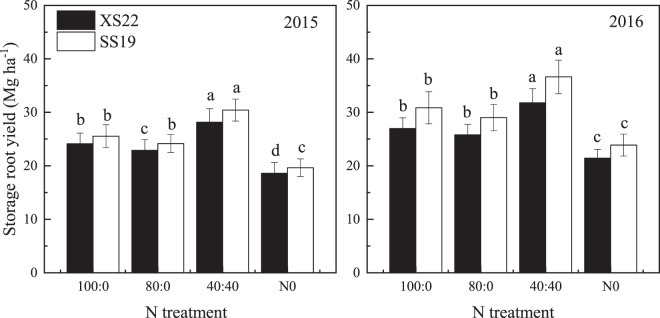
Response of sweetpotato storage root yield of two cultivars (XS22 and SS19) to different N treatments. Different letters indicate statistical difference (*P* < 0.05) among the N treatments in each cultivar.

As shown in Fig. 1, split N application significantly increased storage root yield. In 2015, the storage root yield of the 40:40 treatment was 16.6% (*P* < 0.05) and 19.0% (*P* < 0.05) higher than that of the 100:0 treatment for XS22 and SS19, respectively. In 2016, compared with the 100:0 treatment, the 40:40 treatment had a storage root yield that increased by 17.8% (*P* < 0.05) and 18.6% (*P* < 0.05) for XS22 and SS19, respectively. However, the 80:0 and 100:0 treatments did not differ in yield (*P* > 0.05), except for XS22 in 2015.

### Dynamic simulation of nitrogen uptake in sweetpotato

**Figure 2:**
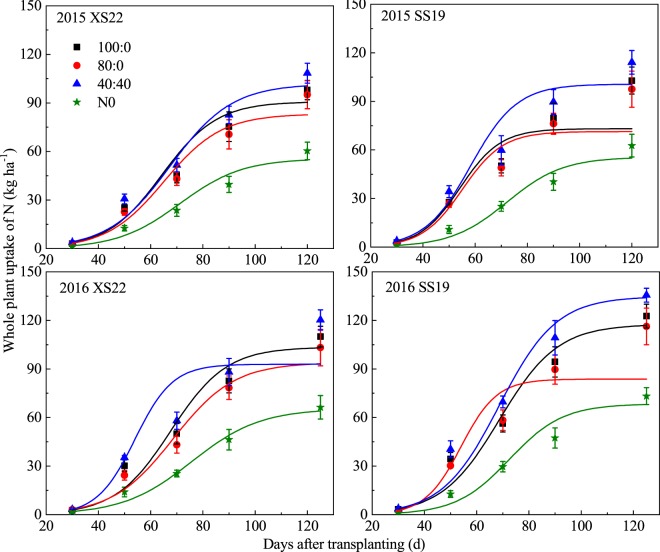
Response of sweetpotato plant N uptake of two cultivars (XS22 and SS19) to different N treatments. Each data point is the mean ± S.E. of three replications.

**Figure 3:**
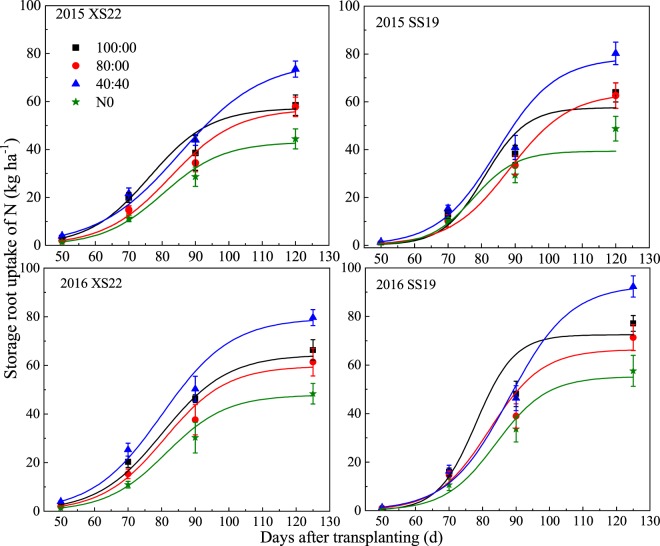
Response of sweetpotato storage root N uptake of two cultivars (XS22 and SS19) to different N treatments. Each data point is the mean ± S.E. of three replications.

N uptake in sweetpotato plant increased from seedling to physiological maturity following a logistic growth curve by days after transplanting (DAT), and there were significant differences among the N treatments (Figs 2 and 3). Compared to the 100:0 treatment, the reduced N rate (80:0) showed a decreased N uptake, but the split N application (40:40) increased the N uptake. As a result, the final N uptake showed a consistent trend for both years with 40:40 > 100:0 > 80:0 > N0.

The N uptake of storage root was increased rapidly after the storage root expansion period (70 DAT), and notable differences were found between different N treatments. The final storage root N uptake ranked as 40:40 > 100:0 > 80:0 > N0 for both years (Fig. 3).

The dynamic uptake of sweetpotato plant N uptake with days after transplanting was simulated using formulas (1)–(2), and differences were observed among the four N treatments (Table 1). During the duration of the fast N uptake phase, the 100:0 treatment had an average rate of 2.06 and 2.02 kg ha^−1^ d^−1^ and an uptake duration of 28.0 and 24.9 d for XS22 and SS19, respectively. Compared to the 100:0 treatment, the 40:40 treatment had an average N uptake rate that increased by 1.0% and 16.1%, respectively, but that of the 80:0 treatment decreased by 9.0% and 16.8% for XS22 and SS19, respectively. The duration of the fast N uptake periods of the 80:0 treatment was slightly different than that of the 100:0 treatment, while that of the 40:40 treatment was 2.0–2.1 d longer than the 100:0 treatment.

**Table 1:** Logistic equation characteristics of the N uptake of the whole plant subjected to different N treatments in 2015 and 2016.

Cultivars	N treatments	Regression equation	R^2^	t_1_ (DAT)	t_2_ (DAT)	T (d)	V_t_ (kg ha^−1^ d^−1^)
**2015**							
XS22	N0	N = 55.6/(1 + 381.7e^−0.0835t^)	0.9118^*^	55.4	87.0	31.6	1.17
	100:0	N = 90.8/(1 + 4434.2e^−0.0945t^)	0.9235^*^	50.3	78.2	27.9	1.92
	80:0	N = 83.2/(1 + 372.4e^−0.0908t^)	0.9154^*^	50.7	79.7	29.0	1.76
	40:40	N = 101.7/(1 + 296.9e^−0.0844t^)	0.9412^*^	51.8	83.0	31.2	2.15
SS19	N0	N = 55.6/(1 + 852.4e^−0.0935t^)	0.9413^*^	58.1	86.3	28.2	1.17
	100:0	N = 73.1/(1 + 1080.0e^−0.1289t^)	0.9310^*^	44.0	64.4	20.4	1.55
	80:0	N = 71.4/(1 + 1185.8e^−0.1284t^)	0.9277^*^	44.9	65.4	20.5	1.51
	40:40	N = 100.7/(1 + 729.8e^−0.1131t^)	0.9457^*^	46.7	70.0	23.3	2.13
**2016**							
XS22	N0	N = 65.5/(1 + 321.7e^−0.0762t^)	0.9657^**^	58.5	93.0	34.5	1.38
	100:0	N = 103.5/(1 + 581.7e^−0.094t^)	0.9254^*^	53.7	81.7	28.0	2.19
	80:0	N = 93.9/(1 + 361.8e^−0.0858t^)	0.9245^*^	53.3	84.0	30.7	1.98
	40:40	N = 99.9/(1 + 503.5e^−0.0912t^)	0.9235^*^	53.8	82.7	28.9	2.00
SS19	N0	N = 68.4/(1 + 1208.3e^−0.0978t^)	0.9344^*^	59.1	86.0	26.9	1.45
	100:0	N = 117.6/(1 + 487.2e^−0.09t^)	0.9266^*^	54.1	83.4	29.3	2.49
	80:0	N = 93.7/(1 + 953.7e^−0.0901t^)	0.9908^**^	61.5	90.8	29.2	1.85
	40:40	N = 134.8/(1 + 536.2e^−0.0865t^)	0.9775^**^	57.4	87.8	30.4	2.56

t: Days after transplanting (DAT); t_1_: Time of whole plant N uptake rate acceleration; t_2_: Time of whole plant N uptake rate deceleration; T: The fast uptake period of whole plant N; V_t_: Average N uptake rate during the fast N uptake period; ^*^significant at *P* < 0.05; ^**^significant at *P* < 0.01.

Differences also existed in the N uptake progress of storage roots (Table 2). In comparison to the 100:0 treatment, the 40:40 treatment had a 0.07–0.23 kg ha^−1^ d^−1^ higher average rate and a 1.6–5.5 d longer of N uptake duration. Furthermore, in comparison with the 100:0 treatment, the 80:0 treatment had −1.1–6.0 d longer N uptake duration but the 0.08 and 0.28 kg ha^−1^ d^−1^ lower average rates for XS22 and SS19, respectively.

**Table 2:** Logistic equation characteristics of the N uptake of the storage root subjected to different N treatments in 2015 and 2016.

Cultivars	N treatments	Regression equation	R^2^	t_1_ (DAT)	t_2_ (DAT)	T (d)	V_t_ (kg ha^−1^ d^−1^)
**2015**							
XS22	N0	N = 43.1/(1 + 7516.1e^−0.1113t^)	0.9802^*^	68.4	92.1	23.7	1.05
	100:0	N = 57.3/(1 + 4432.5e^−0.1101t^)	0.9804^*^	64.3	88.2	23.9	1.38
	80:0	N = 57.0/(1 + 4338.3e^−0.1016t^)	0.9655^*^	69.5	95.4	25.9	1.27
	40:40	N = 76.9/(1 + 2084.3e^−0.0952t^)	0.9823^*^	66.5	94.1	27.7	1.61
SS19	N0	N = 39.4/(1 + 178624.9e^−0.1560t^)	0.9945^**^	69.1	86.0	16.9	1.35
	100:0	N = 57.6/(1 + 10592.0e^−0.15t^)	0.9891^*^	68.4	85.9	17.6	1.89
	80:0	N = 63.8/(1 + 19459.7e^−0.1118t^)	0.9799^*^	76.6	100.1	23.6	1.56
	40:40	N = 78.4/(1 + 15614.2e^−0.1142t^)	0.9584^*^	73.0	96.1	23.1	1.96
**2016**							
XS22	N0	N = 47.9/(1 + 9039.9e^−0.112t^)	0.9896^*^	69.6	93.1	23.5	1.17
	100:0	N = 64.4/(1 + 4028.4e^−0.1035t^)	0.9895^*^	67.5	93.0	25.5	1.46
	80:0	N = 59.8/(1 + 6263.7e^−0.1081t^)	0.9760^*^	68.7	93.1	24.4	1.42
	40:40	N = 79.5/(1 + 2364.3e^−0.097t^)	0.9817^**^	66.5	93.6	27.1	1.69
SS19	N0	N = 55.3/(1 + 61887.3e^−0.1314t^)	0.9990^**^	74.0	94.0	20.0	1.59
	100:0	N = 72.5/(1 + 40389.3e^−0.1307t^)	0.9576^*^	71.1	91.2	20.1	2.08
	80:0	N = 66.5/(1 + 33042.0e^−0.1269t^)	0.9967^**^	71.6	92.4	20.8	1.85
	40:40	N = 93.0/(1 + 15523.0e^−0.1097t^)	0.9641^*^	76.0	100.0	24.0	2.24

t: days after transplanting (DAT); t_1_: Time of storage root N uptake rate acceleration; t_2_: Time of storage root N uptake rate deceleration; T: The fast uptake period of storage root N; V_t_: Average N uptake rate during the fast N uptake period; ^*^significant at *P* < 0.05; ^**^significant at *P* < 0.01.

### Sweetpotato nitrogen uptake and distribution

**Figure 4:**
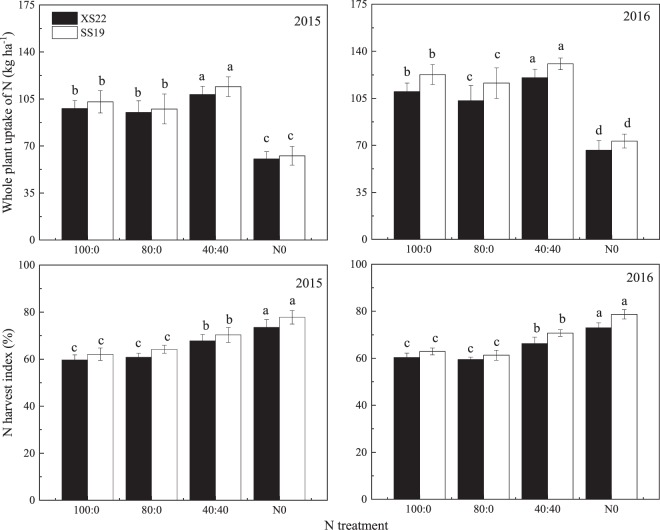
Response of sweetpotato plant N uptake and N harvest index of two cultivars (XS22 and SS19) to different N treatments. Different letters indicate statistical difference (*P* < 0.05) among the N treatments in each cultivar.

Both N uptake and NHI of sweetpotato were significantly affected by N treatments (Fig. 4). The reduced N rate exhibited decreases in N uptake, but split N application noticeably improved sweetpotato N uptake and NHI. Averaged over two years, the total plant N uptake of the 40:40 treatment increased by 10.4 (*P* < 0.05) and 9.6 (*P* < 0.05) g plant^−1^ for XS22 and SS19, respectively, and the NHI increased by 11.7% (*P* < 0.05) and 12.8% (*P* < 0.05), respectively. At the same time, the 100:0 and 80:0 treatments did not differ in N uptake and NHI (*P* > 0.05).

### Nitrogen use efficiency in sweetpotato

In the two experimental years, the NUEs of sweetpotato were significantly (*P* < 0.01) affected by the N treatments (Table 3). Notably, in comparison with the conventional practice application rate, the reduced N rate had an increased partial factor productivity of fertilizer N (PFPN) but a decreased physiological efficiency of fertilizer N (PEN). Split N application dramatically improved NUEs. Compared to the 100:0 treatment, the 40:40 treatment resulted in increases in agronomic use efficiency of fertilizer N (AEN), fertilizer N recovery efficiency (REN), PEN and PFPN of 124.7%, 56.8%, 42.5% and 46.5%, respectively, for XS22, and 122.3%, 51.8%, 46.1% and 47.2%, respectively, for SS19, averaged over two years. Furthermore, a significant genetic difference (*P* < 0.05) was observed between the NUEs of two sweetpotato cultivars in 2016 and between AEN and PFPN in 2015.

**Table 3:** Comparison of the agronomic use efficiency of fertilizer N (AEN), fertilizer N recovery efficiency (REN), physiological efficiency of fertilizer N (PEN) and partial factor productivity of fertilizer N (PFPN) among different N treatments in 2015 and 2016.

Cultivars	N treatments	2015				2016			
		AEN (kg kg^−1^)	REN (%)	PEN (kg kg^−1^)	PFPN (kg kg^−1^)	AEN (kg kg^−1^)	REN (%)	PEN (kg kg^−1^)	PFPN (kg kg^−1^)
XS22	100:0	55.4 b	37.6 c	147.5 b	241.4 c	55.1 b	43.7 b	126.1 b	269.7 c
	80:0	53.6 b	43.4 b	123.7 c	286.1 b	54.3 b	46.1 b	117.7 c	322.5 b
	40:40	119.4 a	60.0 a	198.9 a	351.9 a	128.9 a	67.5 a	190.9 a	397.1 a
SS19	100:0	59.1 b	40.2 b	146.9 b	255.5 c	69.6 b	49.4 b	140.8 b	308.5 c
	80:0	56.4 b	43.6 b	129.3 c	301.9 b	64.1 b	53.9 b	119.0 c	362.8 b
	40:40	134.7 a	64.3 a	209.4 a	380.3 a	151.4 a	71.7 a	211.0 a	450.0 a
**Significance of factors**									
N treatment (N)		427.21^**^	88.41^**^	174.81^**^	96.91^**^	368.51^**^	51.20^**^	248.69^**^	125.36^**^
Cultivar (C)		10.03^*^	2.49^ns^	2.19^ns^	7.78^*^	32.11^**^	8.72^*^	19.91^*^	356.21^**^
N*C		3.14^ns^	0.6^ns^	0.84^ns^	0.42^ns^	1.78^ns^	0.26^ns^	2.99^ns^	3.68^ns^

Means within a column followed by the same letter do not differ significantly at *P* < 0.05. F values and significance levels (^**^P < 0.01, ^*^P < 0.05 and ^ns^*P* ≥ 0.05) are given.

### Correlation coefficients between nitrogen use efficiency and nitrogen uptake

The yield was enhanced with the N uptake of the storage roots at 70–90, 90–120 DAT and total, as well as with N_max_ and T and with the N uptake of the plant at 30–50, 50–70, 70–90, 90–120 DAT and the total, as well as with the N_max_ and T. No significant correlation was observed between the N uptake of storage roots and NUEs at 30–50 and 50–70 DAT. However, the AEN, REN, PEN and PFPN significantly increased with increasing N uptake of the storage roots at 90–120 DAT and total, as well as with T. The AEN and REN significantly increased with the N uptake of the plant at 50–70, 70–90, 90–120 DAT and total, as well as with the N_max_, and T (Table 4). Moreover, the PFPN was significantly correlated with the N uptake of the plant at 50–70, 70–90 DAT and total. These results suggested that too much N uptake at the initial stage of sweetpotato growth inhibited improvements in NUE, while an increase in the N uptake of the storage root and whole plant during the middle and late growth stages was crucial to enhancing NUEs of sweetpotato.

**Table 4:** Correlation coefficients among sweetpotato NUEs and N uptake in four N treatments.

Index		Yield	AEN	REN	PEN	PFPN
N uptake of storage root	30–50 DAT	–0.022	0.271	0.221	0.305	0.091
	50–70 DAT	0.413	0.420	0.399	0.425	0.227
	70–90 DAT	0.769^**^	0.393	0.461	0.295	0.448
	90–120 DAT	0.802^**^	0.804^**^	0.830^**^	0.699^*^	0.870^**^
	Total	0.981^**^	0.881^**^	0.913^**^	0.775^**^	0.871^**^
	N_max_	0.932^**^	0.930^**^	0.943^**^	0.832^**^	0.897^**^
	V_t_	–0.050	0.350	0.317	0.339	0.262
	T	0.648^*^	0.282	0.318	0.239	0.315
N uptake of whole plant	0–30 DAT	0.258	0.215	0.121	0.267	0.055
	30–50 DAT	0.744^**^	0.324	0.423	0.211	0.415
	50–70 DAT	0.703^*^	0.577^*^	0.653^*^	0.444	0.724^**^
	70–90 DAT	0.894^**^	0.587^*^	0.638^*^	0.481	0.639^*^
	90–120 DAT	0.628^*^	0.597^*^	0.618^*^	0.512	0.505
	Total	0.977^**^	0.691^*^	0.790^**^	0.546	0.770^**^
	N_max_	0.783^**^	0.596^*^	0.594^*^	0.526	0.557
	V_t_	–0.042	–0.012	–0.089	0.034	–0.114
	T	0.782^**^	0.595^*^	0.592^*^	0.526	0.554

DAT: Days after transplanting; N_max_: Asymptotic maximum N uptake; V_t_: Average N uptake rate during the fast N uptake period; T: The fast uptake period of sweetpotato N; ^*^significant at *P* < 0.05; ^**^significant at *P* < 0.01.

### Relationship between nitrogen uptake and storage root yield

**Figure 5:**
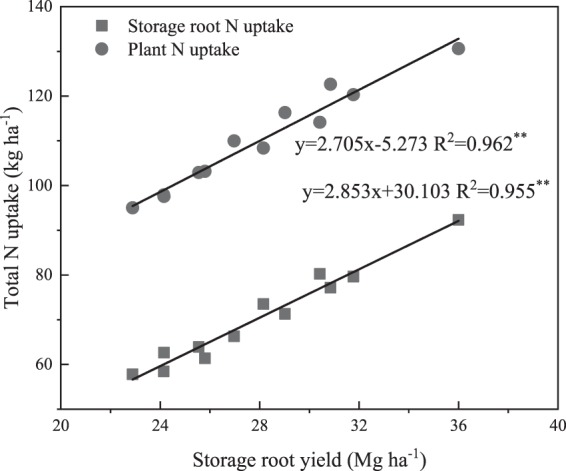
Storage root yield of sweetpotato as a function of N uptake in four N treatments.

Storage root yield was linearly related to the total N uptake of storage roots and whole plants (Fig. 5). A linear regression revealed that the slopes of the regression lines and elevations were significant (*P* < 0.01) for total N uptake in the storage root and whole plant. For every incremental storage root yield, the sweetpotato crop took up 2.71 kg N ha^−1^ from the mineral fertilizer and the soil.

### Economic benefit

In comparison with the 100:0 treatment, the 40:40 treatment showed an obvious increase in output benefit, as shown in Table 5. There were no significant differences in the costs of the inputs (including the costs of labor and fertilizer). Consequently, the net income across both seasons was considerably enhanced by 17.3%–19.9% (849–1093 US$ ha^−1^) in the 40:40 treatment in comparison with that of the 100:0 treatment, according to the real-time price of the local market.

**Table 5:** Economic input, output and net income of sweetpotato production among different N treatments in 2015 and 2016.

Cultivars	N treatments	Output values of storage root yield (US$ ha^−1^)	Fertilizer input (US$ ha^−1^)	Labor input (US$ ha^−1^)	Net income (US$ ha^−1^)
**2015**					
XS22	N0	3986	225		3761
	100:0	5173	284		4889
	80:0	4905	272		4633
	40:40	6032	272	22	5739
SS19	N0	4209	225		3984
	100:0	5475	284		5191
	80:0	5175	272		4903
	40:40	6519	272	22	6226
**2016**					
XS22	N0	4599	225		4374
	100:0	5779	284		5495
	80:0	5529	272		5257
	40:40	6808	272	22	6515
SS19	N0	5119	225		4894
	100:0	6611	284		6327
	80:0	6219	272		5947
	40:40	7714	272	22	7421

## Discussion

The effects of N fertilizer application rate on sweetpotato yield and NUE have been well documented^6,7,10–12^, but the N split and reduced N application for sweetpotato are poorly understood. This study has clearly shown a significant interaction effect of the N rate and application mode on the N dynamics and NUE of sweetpotato. Interestingly, we found that reducing N by 20% under the split application (40:40) did not adversely affect storage root yield but increased yield by 16.6–19.0% compared with the yield under the conventional practice applied N rate. The improved yield by splitting N was probably attributed to the enhanced N nutrition status and improved N uptake and NUE. Previous studies showed that split N application was beneficial to the stability of sweetpotato yield and quality in China^27–29^. This study further confirmed that split N application under a reduced N rate was an important guarantee factor for improving NUE without yield loss. This finding has also provided new insights into the relationship between N uptake and NUE for underground storage root crops, and increasing N uptake in the middle and late stages of crop growth is conducive to higher NUE. These findings could contribute to the further improvement of fertilizer use efficiency.

### Split application improved sweetpotato nitrogen uptake under a reduced nitrogen rate

N is closely related to plant growth and development during the whole growing season. Sweetpotato plants utilize N acquired from applied inputs to produce more storage roots, and N uptake is co–regulated with crop growth^30^. At the early growth stage, sweetpotato plants have a low demand for N nutrient; however, after 35 DAT (tuber initiation stage), sweetpotato needs more nutrient. Splitting the N at the beginning and 35 DAT better satisfied the plant N demand, ensuring an efficient amount of nutrients in the leaves delaying their senescence^27^. This process was accompanied by a long duration and high rate of the rapid N uptake phase, and the N uptake was significantly higher than that in 100:0 treatment in the present study (Fig. 4). On the other hand, the immature root system at the early growth stage limits basal fertilizer N absorption. However, when fertilizer is applied at 35 DAT, the roots of the plants are well developed, and most of the fertilizer N is absorbed within several days after application, resolving the conflict between supply of and demand for N^31^. As a consequence, split N application markedly improved the N uptake of plants during the middle and late growth stages, leading to increased final N uptake and NHI. Similar results have been reported for rice^32^, cotton^33^ and potato^20^. Furthermore, split N enlarged sink capacity^27^, which resulted from the increased storage root weight and storage root number due to their increased N uptake amounts. Thus, split application that synchronizes N application with the N demand of sweet potato plants should allow adequate N absorption to achieve optimum yields under reduced N supply.

### Split nitrogen application improved the nitrogen use efficiency of sweetpotato under a reduced nitrogen rate

N uptake and NUE are mainly affected by N application rates, timing, crop establishment, soil types, irrigation, and climate^34–36^. Excessive N applied at the early growth stage is the main constraint on N uptake and NUE under conventional farmer practices in China^27,34^. The results from this study suggest that both higher yield and NUE may be achieved in sweetpotato production by optimizing N management practices. A reduced 20% N rate with a one-time basal fertilization (80:0) resulted in lower AEN and PEN than that with the conventional application (100:0), but the NUE significantly increased with a reduced 20% N rate under the split application (40:40). This finding indicated that storage root yield decreased in the 80:0 treatment due to the decreased N uptake, AEN and PEN. Consequently, the increased yield under the 40:40 treatment was attributed to the increased N uptake, and NUE. Our results indicated that the REN, AEN, and PFPN were positively related to the N uptake during the middle and late growth stages, which were markedly improved by the split N application, resulting in improved final N uptake, NHI, and NUEs. Based on the above results, we assumed that the N application crop management needed to achieve a greater yield and higher NUE in sweetpotato is primarily associated with split fertilizer N application rather than high N rates.

**Figure 6:**
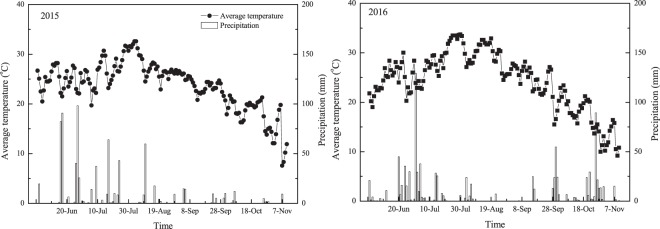
Average air temperature and precipitation over sweetpotato growing season in 2015 and 2016.

Additionally, the summer sweetpotato growth period in China is approximately June to October, with excessively high air temperatures, accompanied by high rainfall starting in late June to mid-July at the tuber initiation stage. The seasonal rainfall distribution was approximately 561.7 and 486 mm between planting and 35 DAT in 2015 and 2016 (Fig. 6). These conditions often lead to a high risk of N leaching events. In our study, a higher sweetpotato N uptake was observed in 2016 than in 2015. The differences were primarily related to the fertilizer loss caused by excessive rainfall in the 2015 early growing season (Fig. 6). Thus, split N application can increase N uptake by reducing leaching and runoff, especially in years with heavy rainfall^37,38^. This study also confirmed that the variable N uptake between years was larger in the 100:0 treatment than in the 40:40 treatment (Fig. 4). Split N at the beginning and 35 DAT can supply sufficient fertilizer timed to maximize crop production without increasing the risk of fertilizer loss to the environment, which was consistent with the results of N fertilizer application in potato production in northeastern Florida^39^.

### Split nitrogen application is a suitable way to improve both yield and nitrogen use efficiency

There is currently challenging to achieve high yield and high N use efficiency in sweetpatoto production. The information gained from this research is valuable for future research and will help develop effective nutrient management strategies. Previous research has shown that conventional N management practices for sweetpotato cause excess vegetative growth, early leaf senescence, low photo assimilation and a low harvest index^7,12,27^. Excessive levels of soil N at early stages of crop development also delay storage root set or bulking^12,40^. N management in sweetpotato should improve the number of effective storage roots and the harvest index by maintaining higher photo assimilation rates during the tuber expansion period. Split N application is recognized as the most promising way to accomplish this goal.

The improve in storage root yield and reduce in fertilizer input resulted in the greatest profit for the split N under a reduced rate. In this research, the corresponding net economic benefit would increase approximately 999 US$ ha^−1^ in average years when sweetpotato was planted with split N application rather than conventional one-time basal fertilization. N splitting is therefore recommended as an alternative approach to synchronously increase the storage root yield, NUE and planting benefit under a reduced N rate in sweetpotato production systems in China. Nevertheless, the percentages of the split should be determined according to the primary soil fertility. The optimal split N regimes may depend on the cultivar or rainfall regime, and this requires further investigations.

## Conclusions

(i) The storage root yield was significantly increased by 16.6–19.0% with a reduced 20% N rate under split application, which was attributed to the enhanced N uptake and NUE.
(ii) Reduced 20% N under the split application increased the sweetpotato N uptake rate and the duration of the rapid N uptake phase, resulting in improved N uptake.
(iii) Split application under a reduced N rate obviously improved the N uptake of sweetpotato during the middle and late growth stages, leading to increased NHI, AEN, REN, PEN and PFPN.
(iv) Split N application with a low N application rate is recommended as an alternative approach to synchronously increase the storage root yield and NUE of the inadequate N management sweetpotato production systems in China.

## Materials and Methods

### Experimental design and crop management

Two field experiments were established at the agronomy research farm of the Agricultural Sciences of Anhui Academy (31°89′N, 117°25′E), Hefei, Anhui, China, in the 2015 and 2016 growing seasons. The soil was loam, containing 11.2 and 10.9 g kg^−1^ organic matter, 0.71 and 0.68 g kg^−1^ total N, 7.6 and 7.9 mg kg^−1^ available phosphorus (P) and 149.5 and 167.3 g kg^−1^ available potassium (K) before sweetpotato transplanting at a soil depth of 0–20 cm in 2015 and 2016, respectively^27^. The rainfall and temperature during two experiment seasons were shown in Fig. 6.

Four N treatments were conducted in the experiments: a conventional N management practice, a single basal application of 100 kg N ha^−1^ (100:0), two treatments that received 20% reduced N (80 kg N ha^−1^) applied either at 100% at basal application (80:0), or two splits of 50% at basal application and 50% at storage root initiation stage (35 DAT, 40:40), and a N omission treatment (N0)^27^. The topdressing N was broadcast at 35 DAT on 23 July 2015 and 24 July 2016. In all treatments, P and K fertilizers (90 kg P_2_O_5_ ha^−1^ equivalent as superphosphate, 150 kg K_2_O ha^−1^ equivalent as potassium sulfate) were applied as basal fertilizer at land preparation. Plots were arranged in a randomized complete block design with three replications. The plot size was 40 m^2^ (5.0 m × 8.0 m) with five rows. Xushu 22 (XS22) and Shangshu 19 (SS19), which are the popular high yielding sweetpotato cultivars in China, were used in the field experiment. XS 22 was long vine type and SS 19 was short vine type, they were typical representative variety promoted and applied in China. Sweetpotato was sown in nutritional beds on 12 April 2015 and 2016, and the sweetpotato seedlings were transplanted on June 18 and June 19 and harvested on 24 October and 6 November 2015 and 2016, respectively. The sweetpotato seedlings were transplanted to the field at a density of 50,000 plants ha^−1^ for the two cultivars. The remainder of the management was based on the high standard of field production.

### Sampling and growth measurement

Five sweetpotato plants per plot were collected at 20-d intervals from July 18 to October 16 in 2015 and from July 19 to October 18 in 2016. Subsequently, sweetpotato plants were separated into four fractions (leaf, petiole and vine, storage root, and root). All samples were dried in a fan-forced oven at 105 °C for 30 min and then at 80 °C for 3 days. The total N content of the different plant organs were determined by Kjeldahl method. Sweetpotato plant N uptake (kg ha^−1^) was calculated based on the biomass weight and the N content. Storage root yields were measured by hand from three central rows in each plot.

### Nitrogen dynamics

To model the N uptake pattern, a logistic model was used to describe the progress of the crop plant N uptake as follows^41,42^:

1 $$N=\frac{{N}_{{\rm{\max }}}}{1+a{e}^{bt}},$$

where, t is the DAT, N (kg ha^−1^) is the N uptake in sweetpotato, N_max_ (kg ha^−1^) is the asymptotic maximum N uptake by sweetpotato, and a and b are the constants to be determined.

The time of the N uptake rate acceleration is t_1_, the time of the N uptake rate deceleration is t_2_, t_2_-t_1_ is the fast uptake period of sweetpotato N, and the average N uptake rate (V_t_) was calculated using the following equations:

From formula (1):

2 $${t}_{1}=-\,\frac{1}{b}\,\mathrm{ln}\,\frac{2+\sqrt{3}}{a},\,{t}_{2}=-\,\frac{1}{b}\,\mathrm{ln}\,\frac{2-\sqrt{3}}{a},\,{V}_{t}=\frac{{N}_{2}-{N}_{1}}{{t}_{2}-{t}_{1}},$$

### Nitrogen use efficiency

The NUEs of the REN, AEN, PFPN and PEN were calculated with the following formulas:

3 $$AEN=\frac{\Delta Y}{{N}_{A}},$$

4 $$REN=\frac{\Delta NU}{{N}_{A}},$$

5 $$PFPN=\frac{Y}{{N}_{A}},$$

6 $$PEN=\frac{\Delta Y}{\Delta {N}_{U}}.$$

The AEN is the increased storage root yield (ΔY) over zero-N plots per unit area of fertilizer N applied (N_A_). The REN is the increased total N uptake over zero-N plots (ΔN_U_). The PFPN is the storage root yield (Y) per unit area of fertilizer N applied (N_A_). The PEN is the increased storage root yield per unit area (ΔY) of increased N uptake over zero-N plots (ΔN_U_)^41^.

### Economic and statistical analysis

The economic analysis was performed based on the cost of inputs and price of produce. Microsoft Excel 2010 and Origin 2018 software were used for data processing and figure drawing, respectively. The variance analysis was performed by Duncan’s new multiple-range test at the 5% probability level in SPSS 20.0 statistical software.
